# The ASK-SEAT: a competency-based assessment scale for students majoring in clinical medicine

**DOI:** 10.1186/s12909-022-03140-0

**Published:** 2022-02-04

**Authors:** Linxiang Huang, Zihua Li, Zeting Huang, Weijie Zhan, Xiaoqing Huang, Haijie Xu, Chibin Cheng, Yingying Zheng, Gang Xin, Shaoyan Zheng, Pi Guo

**Affiliations:** 1 Department of Clinical Medicine, Shantou University Medical College, Shantou, 515041 China; 2 Department of Pediatrics, East Carolina University and Vidant Medical Center, Greenville, NC 27834 USA; 3 Department of Microbiology and Immunology, Shantou University Medical College, Shantou, 515041 China; 4 Department of Higher Medical Education, Shantou University Medical College, Shantou, 515041 China; 5 Department of Public Health and Preventive Medicine, Shantou University Medical College, Shantou, 515041 China

**Keywords:** Competency-based assessment, Self-assessment, Medical education, Licensing examination, Clinical medicine

## Abstract

**Background:**

To validate a competency-based assessment scale for students majoring in clinical medicine, ASK-SEAT. Students’ competency growth across grade years was also examined for trends and gaps.

**Methods:**

Questionnaires were distributed online from May through August in 2018 to Year-2 to Year-6 students who majored in clinical medicine at the Shantou University Medical College (China). Cronbach alpha values were calculated for reliability of the scale, and exploratory factor analysis employed for structural validity. Predictive validity was explored by correlating Year-4 students’ self-assessed competency ratings with their licensing examination scores (based on Kendall’s tau-b values). All students’ competency development over time was examined using the Mann-Whitney U test.

**Results:**

A total of 760 questionnaires meeting the inclusion criteria were analyzed. The overall Cronbach’s alpha value was 0.964, and the item-total correlations were all greater than 0.520. The overall KMO measure was 0.966 and the KMO measure for each item was greater than 0.930 (*P* < 0.001). The eigenvalues of the top 3 components extracted were all greater than 1, explaining 55.351, 7.382, and 5.316% of data variance respectively, and 68.048% cumulatively. These components were aligned with the competency dimensions of skills (S), knowledge (K), and attitude (A). Significant and positive correlations (0.135 < Kendall’s tau-b < 0.276, *p* < 0.05) were found between Year-4 students’ self-rated competency levels and their scores for the licensing examination. Steady competency growth was associated with almost all indicators, with the most pronounced growth in the domain of skills. A lack of steady growth was seen in the indicators of “applying the English language” and “conducting scientific research & innovating”.

**Conclusions:**

The ASK-SEAT, a competency-based assessment scale developed to measure medical students’ competency development shows good reliability and structural validity. For predictive validity, weak-to-moderate correlations are found between Year-4 students’ self-assessment and their performance at the national licensing examination (Year-4 students start their clinical clerkship during the 2nd semester of their 4th year of study). Year-2 to Year-6 students demonstrate steady improvement in the great majority of clinical competency indicators, except in the indicators of “applying the English language” and “conducting scientific research & innovating”.

## Background

In 1978, McGathie et al. prepared a report for the World Health Organization (WHO), advocating for cultivating medical talents through competency-based medical education (CBME) in order to meet the healthcare needs of local populations worldwide [1]. Three decades later, a group of international educators refined CBME as “an outcomes-based approach to the design, implementation, assessment, and evaluation of medical education programs, using an organizing framework of competencies” [2].

Developed countries such as U.K., U.S., and Canada have developed more comprehensive competency-based frameworks [3–6]. For instance, the Accreditation Council for Graduate Medical Education in U.S. expects residents to obtain competencies in 6 areas: patient care, medical knowledge, interpersonal & communication skills, professionalism, practice-based learning & improvement, and system-based practice [4]. The General Medical Council (GMC) in U.K. has outlined, in its Good Medical Practice (GMP), the standards which practitioners shall meet and they span 4 domains: knowledge, skills & performance; safety & quality; communication, partnership & teamwork; and maintaining trust [5]. At CanMEDS 2015, a physician competency framework endorsed by 12 Canadian medical organizations was presented which identified multiple key roles played by a competent physician [6]:

- Medical expert— applying medical knowledge, clinical skills, and professional values to provide quality patient-centered care;
- Communicator—forming relationships with patients and their families which facilitate sharing essential information for the delivery of effective health care;
- Collaborator—working effectively with other health care professionals to provide quality patient-centered care;
- Leader—engaging with others to contribute to realizing visions of quality health care systems;
- Health advocate—contributing expertise and influence to improve healthcare when partnering with communities or patient populations;
- Scholar—demonstrating a commitment to continuous learning and “contributing to the application, dissemination, translation, and creation of knowledge and practices”; and.
- Professional—“being committed to ethical practice, accountability to the profession and society” and maintaining personal health.

In 2014, Sun et al. constructed the Chinese Doctors’ Common Competency Model [7, 8], an initiative jointly approved by the National Medical Examination Center and the Ministry of Education. The model has since served as an important reference and standard for the training of Chinese medical professionals. In July 2017, the General Office of the State Council issued a policy entitled “Deepening the Synergy Between Education and Healthcare System to Further Promote Reforms and Development of Medical Education in China” [9], and highlighted the pressing need to establish a system for the evaluation of medical education.

The medical education in China is administered through a variety of programs. From Year 2 to Year 4, students take courses on medical fundamentals. Year-4 students start their clinical clerkship in the 2nd semester. At Year 5, students attend clinical rotations at teaching hospitals, and receive their bachelor’s degree in medicine at the end of their 5th year of study. Year 6 marks the 1st year of 3 years of standardized resident training. With the “5 + 3” program, students receive both the bachelor’s and master’s degrees when completing their study. With the 8-year track, students are awarded bachelor’s and doctor’s degrees when they graduate.

### National Medical Licensing Examination (NMLE)

In April 2015, the National Medical Examination Center in China reformed the administration of the NMLE into two phases. The Phase-I examination (hereinafter, referred to as “NMLE-Phase I”) contains two sections: basic medical knowledge (hereinafter, referred to as “theory examination”) and basic clinical skills (hereinafter, referred to as “skills examination”), while the Phase-II examination (hereinafter, referred to as “NMLE-Phase II”) tests candidates’ comprehensive clinical knowledge and skills. The clinical skills portion of the examination is modeled after the Objective Structured Clinical Examination (OSCE). Medical students are eligible to take the NMLE-Phase I at the end of their 4th year of study and Phase-II at the end of Year 6 [10]. Unlike most standard tests administered in medical schools, the NMLE evaluates multiple dimensions of candidates’ clinical competency—knowledge and clinical skills—and hence a closer approximation to a more rounded competency-based assessment.

### ASK-SEAT: a competency-based assessment scale

In the 1990s, drawing from the process of cognitive development, George Miller, an American medical educator, proposed “Miller’s Pyramid” for assessing the clinical competencies of medical students and resident physicians [11]. The pyramid illustrates how the ultimate mastery of each competency progresses from the level of cognition to clinical practice, and how different levels of mastery can be measured. The 4 tiers of Miller’s Pyramid comprise the following: 1) Knows (knowledge)—“knows what’s required in order to carry out professional functions effectively”; 2) Knows How (competence)—knows how to use the knowledge acquired (e.g. formulating diagnosis and treatment plans); 3) Shows How (performance)—shows how to perform when facing a patient; and 4) Does (action)—how to act when “functioning independently in a clinical practice”.

**Fig. 1:**
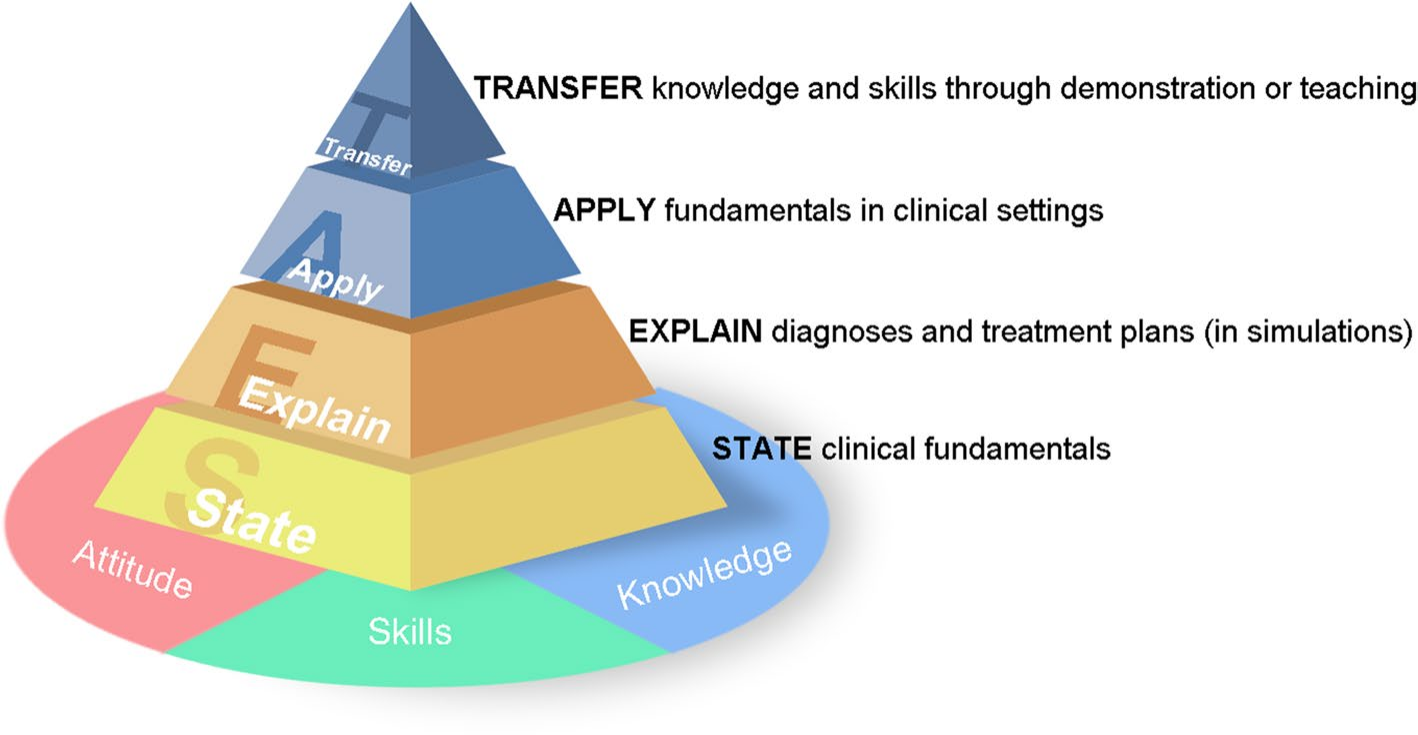
ASK-SEAT: a competency-based assessment scale

However, to the best of our knowledge, there have been few standardized assessment systems, in China or abroad, to evaluate the competency development of students majoring in clinical medicine. Hence, based on the Chinese Doctors’ Common Competency Model created by Sun et al. [7, 8], we created 24 competency indicators for students majoring in clinical medicine which reflect 3 domains of clinicians’ competencies: attitude (A), skills (S), and knowledge (K) (Fig. 1). These indicators broadly reflected the competencies enumerated in the frameworks created in developed countries as illustrated in the above. To enable a more granular assessment of students’ competencies, a matrix design was adopted. Four aspects of mastery—state (S), explain (E), apply (A), and transfer (T)—were used to characterize the 4 levels of competency for each indicator, reflecting the progression of competency in Miller’s Pyramid. A 5-point Likert scale—I (not at all), II (somewhat), III (moderately), IV (mostly), and V (completely)—was added to further quantify each SEAT level. A total of 96 textual descriptions (for 24 indicators and 4 competency levels) were also drafted.

Inspired by Miller’s framework, the tiers of competency in the ASK-SEAT did diverge somewhat from Pyramid, mainly the top 2 tiers. While Miller separated performing in a conditioned setting (“Shows How”) from performing in the real world (“Does”), the ASK-SEAT collapsed these two into one tier (“Apply”) and created an additional tier of “Transfer”. The creation of this tier was underscored by 2 contributing factors related to the mission and focus of medical education in China. First, as presented in the 2015 Global Standards for Quality Improvement: Basic Medical Education by the World Federation for Medical Education (WFME), medical students upon graduation are expected to be able to perform competently the roles of, among others, “teacher” and “scholar” [12]. Second, the ability to transfer prepares medical graduates for “participatory learning” that will be emphasized in the subsequent standardized resident training.

As a pilot study, the ASK-SEAT scale was used in 2018 to assess the core competencies of 155 Year-5 students (“new graduates”) majoring in clinical medicine at the Shantou University Medical College (SUMC) in China [13]. Therefore, the goal of the current study was to validate the results from the pilot study by surveying a larger group of students. Predictive validity of the scale would be tested by correlating students’ self-assessed competency ratings with their performance at the NMLE-Phase I. Participating students’ competency growth across grade years would also be examined for trends and gaps.

## Methods

### Questionnaire

Questionnaires created based on the 24 indicators were distributed via an online platform from May through August 2018 to Year-2 to Year-6 students at the SUMC. A questionnaire response was excluded if it met one of the following criteria: 1) from respondents majoring in clinical medicine at the SUMC but outside the grade years specified; 2) from respondents who supplied identical answers to all questions; 3) from respondents who submitted multiple questionnaires using the same IP address (in this case, only the last questionnaire submitted would be accepted, with the rest, discarded). The questionnaire includes 13 items on personal background and 24 items on competency, all of which are mandatory.

### Data analysis

Statistical analyses were conducted using SPSS 21.0 (IBM Corp. Released 2012. IBM SPSS Statistics for Windows, Version 21.0. Armonk, NY: IBM Corp.). Cronbach alpha values were calculated to examine the reliability of the scale, and exploratory factor analysis (EFA) performed for structural validity. For predictive validity, correlation analysis was carried out (based on Kendall’s tau-b values), using Year-4 students’ NMLE-Phase I scores (consisting of 3 sections: theory, skills, and total) and their self-assessed ASK-SEAT ratings (students’ NMLE-Phase I scores were collected from the Academic Affairs Office of the SUMC). Statistical significance was set at 0.05. The Mann-Whitney U test was employed to identify competency differentials between adjacent grades for students’ competency development over time. Except the correlation analysis which relied solely on information related to Year-4 students, the remaining analyses were carried out using the questionnaires from all participating students.

## Results

### Respondents

Out of 960 questionnaires collected, 760 met the inclusion criteria and were analyzed (366 from female students, accounting for 48.2% of the total). The number of responses in each grade-year group exceeded 150, except the group of Year 6 of slightly more than 100 responses. Participating students’ basic information is summarized in Table 1*.*

**Table 1:** Basic information of questionnaire respondents

Year	Item	Gender			Age				
		Male	Female	Total	18 ~ 20	21 ~ 23	24 ~ 27	Unknown	Total
6	No.	55	49	**104**	*–*	2	102	*–*	**104**
	Percentage	52.90%	47.10%	**100.00%**	*–*	1.90%	98.10%	*–*	**100.00%**
5	No.	80	71	**151**	*–*	64	87	*–*	**151**
	Percentage	53.00%	47.00%	**100.00%**	*–*	42.40%	57.60%	*–*	**100.00%**
4	No.	80	73	**153**	*–*	135	12	6	**153**
	Percentage	52.30%	47.70%	**100.00%**	*–*	88.20%	7.80%	3.90%	**100.00%**
3	No.	91	88	**179**	10	166	1	2	**179**
	Percentage	50.80%	49.20%	**100.00%**	5.60%	92.70%	0.60%	1.10%	**100.00%**
2	No.	88	85	**173**	84	85	*–*	4	**173**
	Percentage	50.90%	49.10%	**100.00%**	48.60%	49.10%	*–*	2.30%	**100.00%**
Total	No.	394	366	**760**	94	452	202	12	**760**
	Percentage	51.80%	48.20%	**100%**	12.40%	59.50%	26.60%	1.60%	**100.00%**

### ASK-SEAT: reliability

The overall Cronbach’s alpha value was 0.964. The item-total correlations were all greater than 0.520, within an acceptable range. Hence, all items were retained, as shown in Table 2.

**Table 2:** Reliability and validity of the ASK-SEAT assessment scale

Competency indicators	Reliability		Factor Loading^a^ after Varimax Rotation		
	Corrected item-total correlation	Cronbach’s alpha if item deleted	Domains		
			S	K	A
S-1 Taking the medical history	.527	.964	**.449**	*–*	.301
S-2 Conducting the physical examination	.616	.963	**.602**	*–*	*–*
S-3 Applying basic operational skills	.706	.963	**.832**	*–*	*–*
S-4 Presenting clinical cases verbally	.776	.962	**.800**	*–*	*–*
S-5 Formulating the treatment plan	.776	.962	**.850**	*–*	*–*
S-6 Ensuring patient safety	.778	.962	**.700**	*–*	.340
S-7 Managing chronic illnesses	.798	.962	**.748**	.302	*–*
S-8 Participating in education & promotion of health	.725	.962	**.564**	*–*	.427
S-9 Conducting emergency rescue	.777	.962	**.785**	*–*	*–*
S-10 Selecting lab tests & medical examinations	.813	.961	**.774**	.324	*–*
K-1 Understanding the healthcare system	.775	.962	**.642**	.464	*–*
K-2 Retrieving, organizing, & analyzing medical information	.727	.962	.364	**.734**	*–*
K-3 Applying the English language to knowledge acquisition, professional exchange & clinical practice	.567	.964	*–*	**.781**	*–*
K-4 Acquiring & applying basic biomedical knowledge	.711	.962	.387	**.701**	*–*
K-5 Acquiring & applying knowledge of social science	.767	.962	.445	**.615**	.314
K-6 Acquiring & applying clinical knowledge	.845	.961	**.646**	.469	.336
K-7 Updating knowledge and skills	.718	.962	*–*	**.639**	.459
K-8 Applying critical thinking	.730	.962	*–*	**.664**	.484
K-9 Conducting scientific research & innovating	.717	.962	.311	**.737**	*–*
A-1 Controlling patient’s medical expenses	.703	.963	**.543**	.402	*–*
A-2 Maintaining psychological health	.655	.963	*–*	*–*	**.719**
A-3 Communicating & cooperating with clients	.744	.962	.406	*–*	**.727**
A-4 Protecting patient confidentiality	.578	.964	*–*	*–*	**.821**
A-5 Teamwork	.601	.963	*–*	*–*	**.787**

^a^The factor loading refers to the correlation coefficient between the indicator and the relevant domains, ranging from 0 (weakest) to 1 (strongest). Indicators with a factor loading below 0.3 are excluded from the table

### ASK-SEAT: structural validity & predictive validity

EFA based on varimax rotation was first performed without a limit to the number of factors to be extracted. The data variance explained by the 4 factors extracted was 55.351, 7.382, 5.316, and 4.523% respectively, and 72.572% cumulatively. In this round, only 2 indicators loaded on the 4th factor: taking the medical history, and conducting the physical examination. Hence, a 2nd round of EFA was performed where the number of factors to be extracted was limited to 3. The 2nd EFA yielded a linear correlation among the variables (24 items) and an adequate data structure (overall KMO = 0.966; KMOs for items > 0.930; *P* < 0.001). Hence, principal component extraction was deemed suitable. The eigenvalues of the top 3 components extracted were all greater than 1 (explaining 55.351, 7.382, and 5.316% of data variance respectively, and 68.048% cumulatively). These components corresponded to the 3 competency dimensions of skills (S), knowledge (K), and attitude (A). Three indicators were not aligned as expected: K-1 (“understanding the healthcare system”), K-6 (“acquiring & applying clinical knowledge”), and A-1 (“controlling patient’s medical expenses”). After taking into consideration the grade-specific results where selected indicators were also aligned differently, a decision was made not to make further adjustment and to retain the initial alignments of these 3 indicators to maximize the utility of the scale (Table 2).

**Fig. 2:**
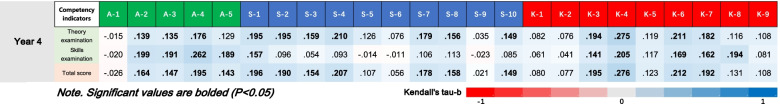
Correlations between Year-4 students’ self-assessed competency levels and their scorings for the NMLE-Phase I

The correlation between Year-4 students’ self-assessed ASK-SEAT ratings and their performance at the NMLE-Phase I is presented in Fig. 2 where significant correlations are in bold. Significant and positive correlations (0.135 < Kendall’s tau-b < 0.276, *P* < 0.05) spread generally evenly across 3 domains of attitude (A), skills (S), and knowledge (K) for the theory as well as the combined total portion (theory plus skills). In the skills portion, more correlations were associated with the domains of attitude (A) and knowledge (K).

### Competency growth

**Fig. 3:**
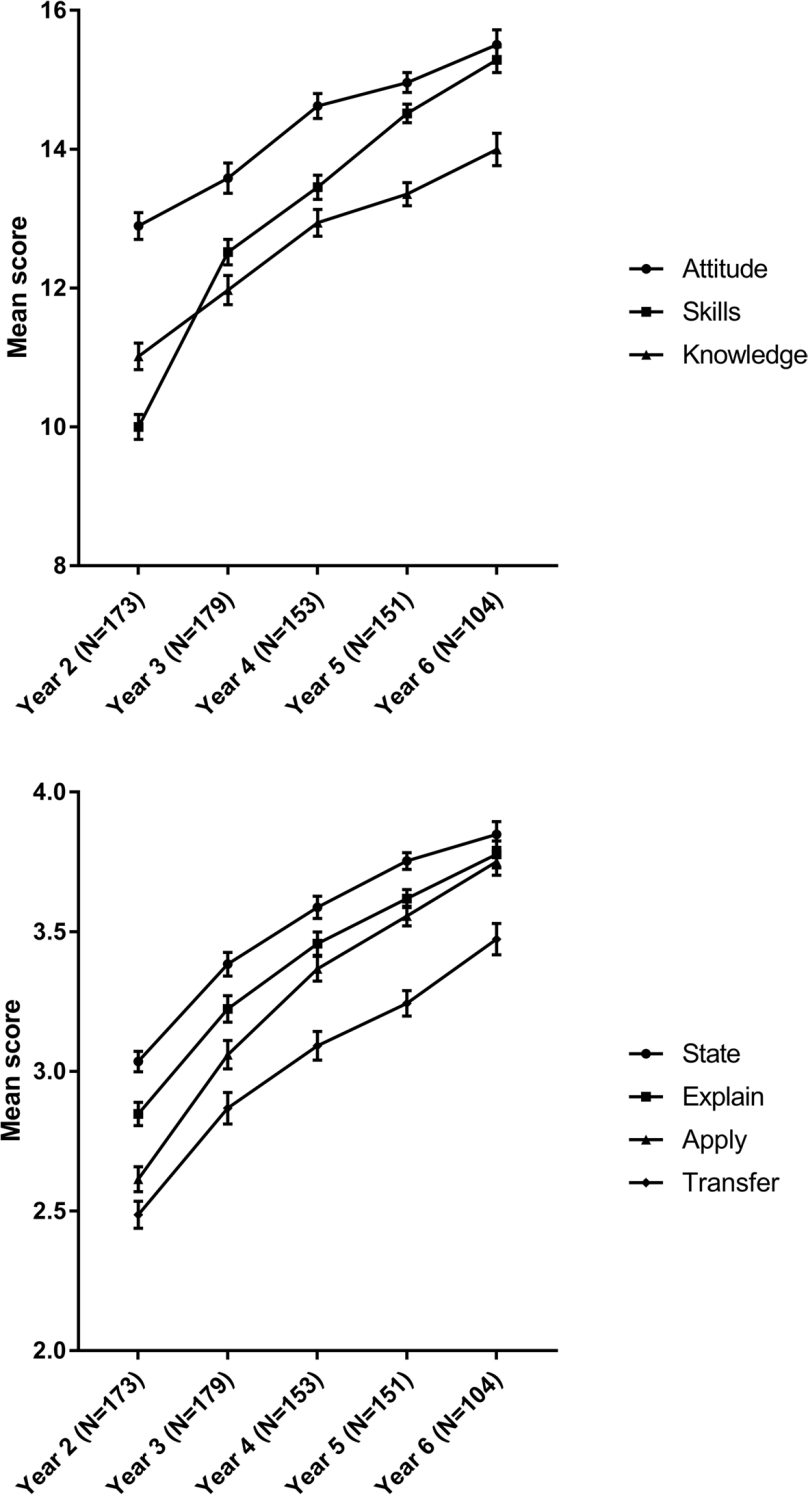
-1 Competency growth by domain—attitude (A), skills (S), and knowledge (K)—among Year-2 to Year-6 students. 3–2. Competency growth by competency level—state (S), explain (E), apply (A), transfer (T)—among Year-2 to Year-6 students

The mean ratings (with standard errors) of competency by students (Year-2 to Year-6) are graphed in Fig. 3-1 (by domain) and Fig. 3-2 (by competency level). The highest rating for each grade year was in the domain of attitude (A), and the most improvement was in the domain of skills (S) (Fig. 3-1). For the level of competency, students’ performance trended steadily upward across grade years, with the highest rating associated with the level of “state” followed by the levels of “explain”, “apply”, and “transfer”, in that order and for each grade year (Fig. 3-2).

**Fig. 4:**
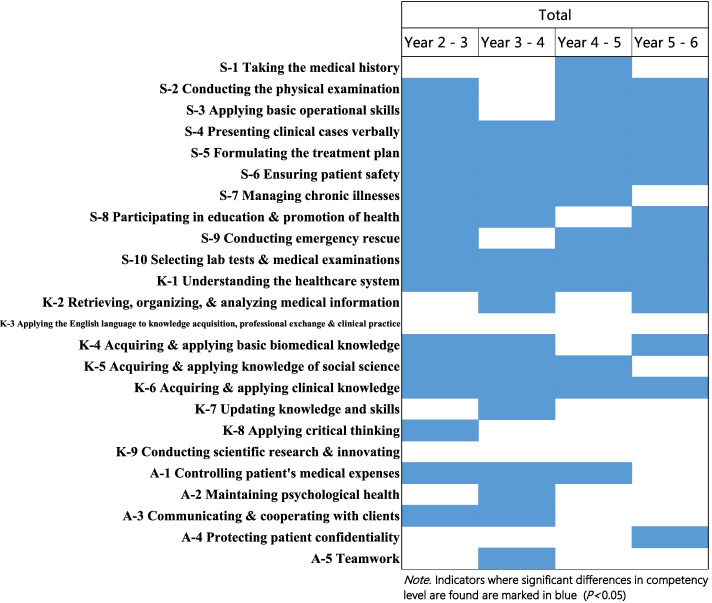
Significant competency growth between adjacent years (Year-2 to Year-6) by indicator based on the Mann-Whitney U test

The indicators with significant and positive changes in competency level between adjacent grades are marked in blue in Fig. 4. Growths were reported by students for all indicators except 2, with the most pronounced growth in the domain of skills. The 2 indicators where no steady growth was seen were “applying the English language” and “conducting scientific research & innovating”.

## Discussion

### ASK-SEAT: a competency-based assessment scale

The overall Cronbach’s alpha value (0.964) and the item-total correlations (all greater than 0.520) demonstrated good reliability of the ASK-SEAT scale. The three factors extracted in the pilot study—attitude (A), skills (S), and knowledge (K)—were also confirmed through EFA. Meanwhile, as presented in Table 2, most indicators had loadings of more than .30 (the cutoff) on 2 and sometimes 3 of the factors extracted. The mastery level of one dimension of a competency can have an additive effect on the mastery level of another dimension. For example, a more clinically-skilled student is likely to give a higher rating for his/her mastery level in *both* knowledge and skills dimensions, since knowledge serves as the foundation of skills and, as skills are developed, the relevant knowledge is also enforced. Hence, a less “clean” loading between indicators and factors could be the reflection of this unique property of competency. If the loading cutoff were raised (to higher than .30), we would have obtained a cleaner set of loadings. However, the unique additive effect between competency dimensions would have also been masked. Further exploration of this topic in future studies may help shed more illuminating lights.

In the current study, positive correlations between Year-4 students’ self-assessed competency ratings and their scorings for the *theory-knowledge portion* of the licensing examination were found in 14 (out of 24) indicators, and the correlations spread generally evenly across the 3 domains (attitude, skills, knowledge). Positive correlations between students’ self-assessment and their performance in the *skills portion* of the examination were also seen in 10 indicators, but only 1 correlation pertained to the “skills domain” (i.e. S1: Taking the medical history). To ace the skills section of the examination (modeled after the OSCE), students needed to draw from their capabilities in all 3 domains. Students’ strong foundation in “attitude” and “knowledge” domains (as evidenced in their scoring for the theory-knowledge portion of the NMLE) contributed meaningfully to their overall scoring in the skills portion. On the other hand, the correlation in only 1 indicator which pertained to the skills domain might be attributed to the fact that Year-4 students just started their clinical clerkship during the 2nd semester of their 4th year of study (who would receive additional clinical exposure and training during subsequent clinical rotations during Year 5 and resident training from Year 6 through Year 8). Therefore, it is not entirely surprising that the correlation between the self-assessment and the skills portion skewed toward the domains of “attitude” and “knowledge”.

At the same time, the correlations ranged from weak to moderate, even though they met the statistical significance set for the study. In order to more definitively characterize how medical students’ self-assessed competencies correlate with their performance in the licensing examination, follow-on studies can replicate the correlation analysis (proposed in this study) among students of more advanced grade years (i.e. Year 5 to Year 8) as well as between students’ self-assessed ratings and their scorings for the NMLE-Phase II taken at the end of Year 6 (which evaluates candidates’ *comprehensive* knowledge and clinical skills). By then, students will have accumulated more clinical experience and are more *cognitively* equipped to rate their clinical skills levels. Different correlation patterns could well emerge in these investigations. Nevertheless, the positive correlations between students’ self-assessed competencies in “attitude” and “knowledge” domains and their scorings for both the theory and overall skills portions of the NMLE did testify to the SUMC’s investment in building students’ capabilities in these 2 domains.

In China, emphasis has been increasingly placed on physicians’ professionalism as well as clinical skills—dimensions vividly captured by the ASK-SEAT which measures multiple domains. The 24 indicators in the ASK-SEAT scale can thus serve as a more detailed reference to assist fine-tuning and redesigning the NMLE, so the sensitivity of the licensing examination as an assessment process can be enhanced.

During our research, we did locate a system in Germany which was designed by Prediger et al. to assess the competencies of medical students [14]. While both the ASK-SEAT and the system developed by Prediger et al. are drawn from the framework of Miller and examine competencies beyond knowledge, the two systems differ in a number of aspects. First, the system by Prediger et al. targets students of advanced grade years (i.e. Year-5 and Year-6 students in a 6-year curriculum which consists of 2 years of pre-clinical and 3–4 years of clinical exposure) by simulating the 1st working day of a resident in a hospital. The ASK-SEAT, at least for the current study, has been conducted among a broader group of students, including those in their earlier years of study (Year-2 to Year-6). Second, the system by Prediger et al. aims at a more fine-tuned and deeper assessment of competencies of a more targeted group of students. The ASK-SEAT is, on the other hand, a standardized tool that requires less time and resources to perform and can be administered to a larger and more diverse set of students. Third, contrasted with the ASK-SEAT which is an assessment *scale*, the system by Prediger et al. contains a *checklist* of competencies and each indicator is measured using different instruments (e.g. questionnaire, case vignette). Fourth, the system by Prediger et al. focuses more on applying “generic” skills in a clinical setting (e.g. teamwork and collegiality; structure, work planning, and priorities; scientifically and empirically grounded method of working; and verbal communication with colleagues and supervisors). The ASK-SEAT stresses, instead, on competencies specific to clinical medicine (Table 2).

### Competency development continuum & discriminating competencies

Steady improvements in all 3 competency domains were seen across grade years, with an accelerated improvement in the domain of skills (Fig. 3-1). At the SUMC, the curriculum of “Basic Clinical Skills” is taught to students of Year 2 to Year 3. More importantly, students start clinical clerkship in the 2nd semester of Year 4, before advancing to Year 5 when they are exposed to more clinical practice on the rotation basis. From Year 6 onward, students start receiving resident training which will last for 3 years. Students’ increasing exposure to clinical practice from Year 4 to Year 6 might have thus contributed to the accelerated growth trajectory in the domain of skills.

Furthermore, as shown in Fig. 3–2, the gaps also grew narrower between the competency level of “apply” and the levels of “state” and “explain” from Year 3 to Year 6, indicating a *faster* improvement in students’ ability to apply what they acquired. In an invited review published in 1990 where Miller presented his framework of Pyramid [11], he noted that the higher tiers of competencies (“Shows How” and “Does”) might “imply” the mastery of the infrastructure level of competencies (“Knows” and “Knows How”). The narrowing gap portrayed in Fig. 3–2 appears to provide empirical evidence to support this reasoning. Students’ increasing ability to apply could be attributable to not only more hands-on opportunities (through clinical clerkship and rotations), but also potentially the cumulative mastery of “stating” and “explaining” over time (gains from competency acquisition require time to come to full fruition). Follow-on research can further validate these attributions.

On the other hand, students also reported a lack of steady growth in 2 indicators: “applying the English language” and “conducting scientific research & innovating”. Interestingly, these 2 indicators were also the discriminating competencies identified in an earlier study—competencies which differentiated the high-performer from the typical-performer [15], although the discriminating indicators found in that study were derived only from Year-5 students (students receive a bachelor’s degree at the end of the 5th year—the 1st milestone in their medical study). Future studies can explore discriminating competencies during different “milestone” years, for example, Year 3 (before students start receiving formalized clinical exposure) and Year 8 (when students complete the full length of their study). The insights uncovered can be converted to actionable strategies to augment the current curriculum, so students can be better prepared to master not only the clinical fundamentals but also capabilities that will catapult them to becoming high-performers.

Future research can also test the ASK-SEAT scale among students of advanced grade-years, particularly those who are more deeply immersed in the resident training, to contrast and compare with the current findings derived from students in earlier years of their study.

### Implications

Given the scant availability, in China and abroad, of standardized competency-based assessment measures to gauge the progress of students majoring in clinical medicine through their education, the development and validation of the ASK-SEAT scale offer valuable learnings. The ASK-SEAT is relatively straightforward and less time- and resource-intensive to implement, and can also be modified in accordance with particular competency requirements by individual medical education institutes. Its applicability among not only the Chinese medical students is supported by the scale mirroring the competency frameworks endorsed by governing institutes outside China and in developed countries (as referenced earlier in Background of this report) and the broadly recognized Miller’s Pyramid. In the meantime, Miller’s model was expanded in the ASK-SEAT to include a competency level of “transfer”. This top layer of competency captures the spirit of the role of “scholar” declared at CanMEDS, the role of teaching, training, and mentoring expected of a practitioner in the GMC’s GMP, and the role of “educating patients, families, students, residents, and other health professionals” outlined in the ACGME’s competency framework.

As a tool that is less elaborate to implement (compared to, for instance, the system by Prediger et al.) and validated across multiple grade years, the ASK-SEAT can be integrated into formative assessment of a diverse base of medical students to facilitate more frequent “check-ins” of students’ ongoing development through their years of study. The scale can be completed by students themselves (self-assessment) or by other stakeholders with a vested interest in medical education (such as instructors and supervising physicians).

### Study limitations

Due to time and manpower constraints, self-assessment was sampled from students of 5 grade years as proxies to measure the competency development continuum. A longitudinal follow-up of same groups of students over a longer period of time (as students gain more confidence from additional coursework and clinical rotations) will be needed to confirm the findings from the current study. Secondly, there was no input collected from stakeholders such as instructors, supervising physicians, and student peers to corroborate students’ self-assessment.

## Conclusions

The ASK-SEAT, a competency-based assessment scale developed to measure medical students’ competency development, shows good reliability and structural validity. For predictive validity, weak-to-moderate correlations are found between Year-4 students’ self-assessment and their performance at the national licensing examination (Year-4 students start their clinical clerkship during the 2nd semester of their 4th year of study). Year-2 to Year-6 students also demonstrate steady improvement in the great majority of clinical competency indicators measured, except in the indicators of “applying the English language” and “conducting scientific research & innovating”.

## Data Availability

The datasets generated and analyzed during the current study are available from the corresponding authors on reasonable request.

## Abbreviations

WHO: World Health Organization
CBME: Competency-based medical education
SUMC: Shantou University Medical College
NMLE: National Medical Licensing Examination
EFA: Exploratory factor analysis
